# Lymphocyte subsets and inflammatory factors as predictors of immunotherapy efficacy in patients with hepatocellular carcinoma

**DOI:** 10.1038/s41598-023-49810-x

**Published:** 2023-12-18

**Authors:** Gao Shuyue, Cheng Jiamin, Qian Niansong

**Affiliations:** 1 The First Medical Center of Chinese PLA General Hospital, Beijing, 100853 China; 2 Department of Hepatology, the Fifth Medical Center of Chinese PLA General Hospital, Beijing, 100853 China; 3 Department of Respiratory, the Eighth Medical Center of Chinese PLA General Hospital, Beijing, 100853 China; 4 Department of Oncology, Hainan Branch of Chinese PLA General Hospital, Sanya, 572000 Hainan China

**Keywords:** Hepatocellular carcinoma, Gastrointestinal cancer

## Abstract

We aimed to investigate the correlation between lymphocyte subpopulations expressing inhibitor receptors, IL-6 levels, and the efficacy of immunotherapy in patients with hepatocellular carcinoma. Blood samples were prospectively collected before and after immunotherapy from patients with intermediate and advanced hepatocellular carcinoma who were treated with immunotherapy at the Fifth Medical Center of the PLA General Hospital from August 2022 to October 2023. According to the efficacy of the patients, patients were divided into effective and ineffective groups, with 40 in the effective group and 44 in the ineffective group. We compared changes in lymphocyte subsets and IL-6 levels between the two groups. Optimal cut-off value was determined using ROC curves. Then, patients were categorized into high and low groups based on cut-off value, and the disease control rates and progression free survival were compared. Before immunotherapy, there were no significant differences in the baseline levels of lymphocyte subsets (PD1 + TIM3 + T/T, TIGIT + T/T, TIM3 + T/T, CTLA4 + T/T, LAG3 + T/T, PD1 + T/T) and IL-6 between the two groups (*P* > 0.05). After immunotherapy, the levels of PD1 + TIM3 + T/T, TIGIT + T/T, and IL-6 in the effective group were lower than those in the ineffective group and these differences were statistically significant (*P* = 0.001, *P* = 0.008, *P* = 0.000). However, the levels of other lymphocyte subsets showed no significant difference. Using the ROC curve to assess efficacy prediction, PD1 + TIM3 + T/T, TIGIT + T/T and IL-6 demonstrated high predictive ability (AUC = 0.79, AUC = 0.81, AUC = 0.78). The predictive value of efficacy was further improved when all three factors were combined (AUC = 0.92, *P* = 0.000). Based on the ROC curve, we identified optimal cut-off value for three factors. Notably, patients with values below the optimal cut-off value had higher disease control rate and progression free survival. The levels of PD1 + TIM3 + T/T, TIGIT + T/T, and IL-6 after 2 cycles of immunotherapy may serve as predictors of treatment efficacy in patients with hepatocellular carcinoma.

## Introduction

Liver cancer stands as one of the most prevalent tumors worldwide, notably, China, bearing a high burden of hepatitis B infections, has the highest incidence and mortality rate for liver cancer globally^1^. The advent of immunotherapy has provided new opportunities to inhibit tumor progression, recurrence and metastasis. However, in clinical settings, its therapeutic efficacy doesn’t always mirror the promising results seen in large clinical studies, due to individual patient variations. Additionally, concerns have arisen regarding immune-related adverse side effects and new challenges like drug tolerance and immune escape. It has become a hot issue to predict the efficacy of immunotherapy and explore the indicators with high predictive value. An increasing number of studies have showed that various immune checkpoint receptors, such as PD-1, CTLA4, TIGIT, TIM3, LAG3, and inflammatory factors, are expressed and altered in the tumor microenvironment. Importantly, these alterations might be closely correlated with the treatment efficacy^2^. In mouse models of melanoma, colon cancer, and breast cancer, Sakuishi et al. found that CD8+ T cells co-expressing PD1+ and TIM3 + CD8+ T accounted for the majority of the total T cell population. After simultaneous blockade of these two immunosuppressive receptors, there was an increase in cytokines with tumor-killing effects compared to the previous period, thus partially restoring T cell functions^3^. In the same year, Fourcade et al. validated these results in patients with melanoma and also found that PD1 + TIM3 + CD8+ T cells had poorer function and produced lower levels of IFN-γ, TNF, and IL-2, than TIM3-PD1+ and TIM3-PD1-CD8+ T cells. Furthermore, simultaneous blockade of TIM3 and PD1 T cell exhaustion/dysfunction could be reversed^4^. Additionally, in patients with advanced gastric and colorectal cancers, shorter survival was observed when PD1 + TIM3+ cells constituted a higher proportion of total T cell population^5,6^. Thus, we selected lymphocyte subpopulations expressing PD1, CTLA4, TIGIT, TIM3, LAG3, and those co-expressing PD1 and TIM3, and IL-6 were selected for the present study.

## Methods

### Patients

Blood samples were prospectively collected before and after 2 cycles of immunotherapy from patients with intermediate and advanced hepatocellular carcinoma who were treated with immunotherapy at the Fifth Medical Center of the General Hospital of the People's Liberation Army (PLA) from August 2022 to October 2023. Inclusion criteria: aged ≥ 18 years; Pathologically confirmed diagnosis of hepatocellular carcinoma; BCLC stage B and C; Eastern Cooperative Oncology Group (ECOG) of 0–1; liver function with Child–Pugh A or B; Patients received immune checkpoint inhibitors following the standard dosing and schedules recommended by NCCN guidelines and drug instructions (Pembrolizumab or Sintilimab 200 mg Q3W + lenvatinib 8 mg/12 mg po qd); have at least one measurable lesion according to Response Evaluation Criteria in Solid Tumors (RECIST) version 1.1; Patients who have not received prior immunotherapy, allowed for receive two or more cycles of immunotherapy, and have completed at least one efficacy evaluation. Exclusion criteria: Patients with severe infection or discontinued immunotherapy; Patients who have recently undergone interventional and ablative therapy.

### Methods

Blood samples were collected the day before receiving the first dose of immunotherapy and the day before receiving the third cycle of immunotherapy from patients who had not previously received immunotherapy. Efficacy was assessed at least once. For those who did not visit the hospital monthly for efficacy evaluation after 2 cycles of treatment, telephone follow-up was used, which was conducted before the next cycle of immunotherapy according to their specific medication cycle time. The collected blood samples were used for sorting and counting of lymphocyte subpopulations and measuring IL-6 levels by flow cytometry and ELISA techniques.

Methods of flow cytometry: each tube added peripheral blood specimens 100 uL; the tube added antibody CD3-cFluor BV421, PD1-PE-Dazzle594, TIM3-BV785, TIGIT-PE, CTLA4-PE-Cy7, LAG3-Alexa Fluor488 and shook for 5 s, and incubated for 15 min in the dark. 3 mL of hemolysin containing fixative was added into all tubes, shaking for 10 s, and then incubated for 5 min away from light; Centrifuge 800 g at room temperature for 5 min, discarded the supernatant and add 1 mL normal saline, shook the suspended cells, then added 4 mL normal saline, centrifuged 800*g* at room temperature for 5 min, discarded the supernatant and absorbed the remaining liquid with tissue; added 100 uL normal saline to the suspended cells, and then load the machine.

All methods were carried out in accordance with relevant guidelines and regulations. This research was approved by the Ethics Committee of the Fifth Medical Center of the General Hospital of the PLA and the patients/participants provided their written informed consent to participate in this study.

### Outcomes and assessments

Tumor assessment by computed tomography or magnetic resonance imaging was conducted at baseline, every 6 weeks. Treatment efficacy was assessed according to RECIST 1.1 criteria. Histologic grade was based on EdmondSon-Steiner. The portal vein invasion determined by radiological examinations.

The patients with PD were classified as the ineffective group and the rest as the effective group. Lymphocyte subsets and IL-6 were compared before and after treatment. Predictive value: With disease control as the state variable and lymphocyte subsets and IL-6 after treatment as the test variable, ROC curves were drawn to predict the efficacy of ICIs and divided into high and low groups according to the optimal cut-off value. Survival analysis was performed to compare the difference in DCR and PFS between the high- and low-value groups.

The primary endpoints were PFS (time from randomization to disease progression per independent review facility [IRF]-assessed RECIST 1.1 or death from any cause, whichever occurred first) and DCR. Complete remission (CR): complete disappearance of all target lesions and reduction of pathologic lymph nodes to the normal range. Partial response (PR): ≥ 30% reduction in the sum of diameters of all measurable target lesions. Stable disease (SD): between partial remission and disease progression. Disease progression (PD): ≥ 20% increase in the sum of diameters of all measurable target lesions or the appearance of new lesions. DCR = CR + PR + SD/total.

### Statistical analysis

Data were analyzed using SPSS Statistics 26 software. Descriptive statistics were presented as mean ± standard deviation or median and interquartile range. Independent t-tests were used for comparing two independent data, and non-parametric tests were used for non-normal distribution. The predictive value was analyzed using the ROC curve and the area under the curve (AUC). The ROC curve was used to determine the optimal cut-off value. The Kaplan–Meier technique was used for survival analysis and compared using the log-rank test. Statistical differences were considered to exist at *P* < 0.05.

## Results

### Baseline and clinical characteristics

The baseline characteristics of the 84 patients included in this study were shown in Table 1. After 2 cycles of treatment, 1 patient had CR, 10 had PR, 29 had SD, and 44 had PD. Patients with CR, PR, and SD were categorized as the effective group, and those with PD were categorized as the ineffective group. There were 40 patients in the effective group and 44 patients in the ineffective group. The two groups showed no statistically significant difference in age, gender, Child–pugh score, ECOG status, extrahepatic spread, portal vein invasion, and other baseline clinical characteristics (*P* > 0.05). The correlations between the IL-6 and Lymphocyte subsets levels were analyzed (Spearman correlation analysis), shown in Table 2. The correlations between the PD1 + TIM3+/T, TIGIT + T/T and IL-6 showed no differences.

**Table 1:** Baseline characteristics.

Characteristics	Effective (%)	Ineffective (%)	T/*X*^2^	*P*
Age (Mean)	54.4	58.5		
Gender/N (%)				
Male	31 (77.50%)	30 (66.7%)	1.23	0.27
Female	9 (22.5%)	15 (33.3%)		
Smoking/N (%)				
Yes	27 (67.5%)	33 (73.3%)	0.029	0.864
No	13 (32.5%)	12 (26.7%)		
ECOG PS/N (%)				
0	25 (62.5%)	36 (80%)	3.2	0.07
1	15 (37.5%)	9 (20%)		
Child–Pugh				
A	28 (70%)	31 (68.9%)	0.012	0.912
B	12 (30%)	14 (31.1%)		
BCLC stage				
B	17 (42.5%))	17 (37.8%)	0.197	0.657
C	23 (57.5%)	28 (62.2%)		
Histologic grade				
Well differentiated	6 (15%)	9 (20%)	1.276	0.528
Moderately differentiated	27 (67.5%)	25 (55.6%)		
Poorly differentiated	7 (17.5%)	11 (24.4%)		
Size				
< 5 cm	15 (37.5%)	16 (35.6%)	0.035	0.853
≥ 5 cm	25 (62.5%)	29 (64.4%)		
Extrahepatic spread				
Yes	16 (40%)	20 (44.4%)	0.171	0.679
No	24 (60%)	25 (55.6%)		
Portal vein invasion				
Yes	29 (72.5%)	32 (71.1%)	0.02	0.887
No	11 (27.5%)	13 (28.9%)		
AFP (ng/mL)				
< 400	22 (55%)	18 (40%)	1.912	0.167
≥ 400	18 (45%)	27 (60%)

**Table 2:** The correlations between lymphocyte subsets and IL-6.

	TIM3 + T/T	PD1 + T/T	CTLA4 + T/T	LAG3 + T/T	PD1 + TIM3+/T	TIGIT + T/T	IL-6
TIM3 + T/T	1	0.937**	0.721**	0.944**	− 0.263	− 0.098	0.813**
PD1 + T/T	0.937**	1	0.751**	0.927**	− 0.082	− 0.086	0.83**
CTLA4 + T/	0.721**	0.751**	1	0.713**	0.006	− 0.17	0.605**
LAG3 + T/T	0.944**	0.927**	0.713**	1	− 0.125	− 0.121	0.827**
PD1 + TIM3+/T	− 0.263	1.927	0.006	− 0.125	1	0.28	0.196
TIGIT + T/T	− 0.098	2.927	− 0.17	− 0.121	0.28	1	− 0.05
IL-6	0.813**	3.927**	0.605**	0.827**	0.196	− 0.05	1

**Correlation is significant at the 0.01 level (2-tailed).

### Lymphocyte subsets and IL-6 comparison between the effective and ineffective groups before immunotherapy

**Figure 1:**
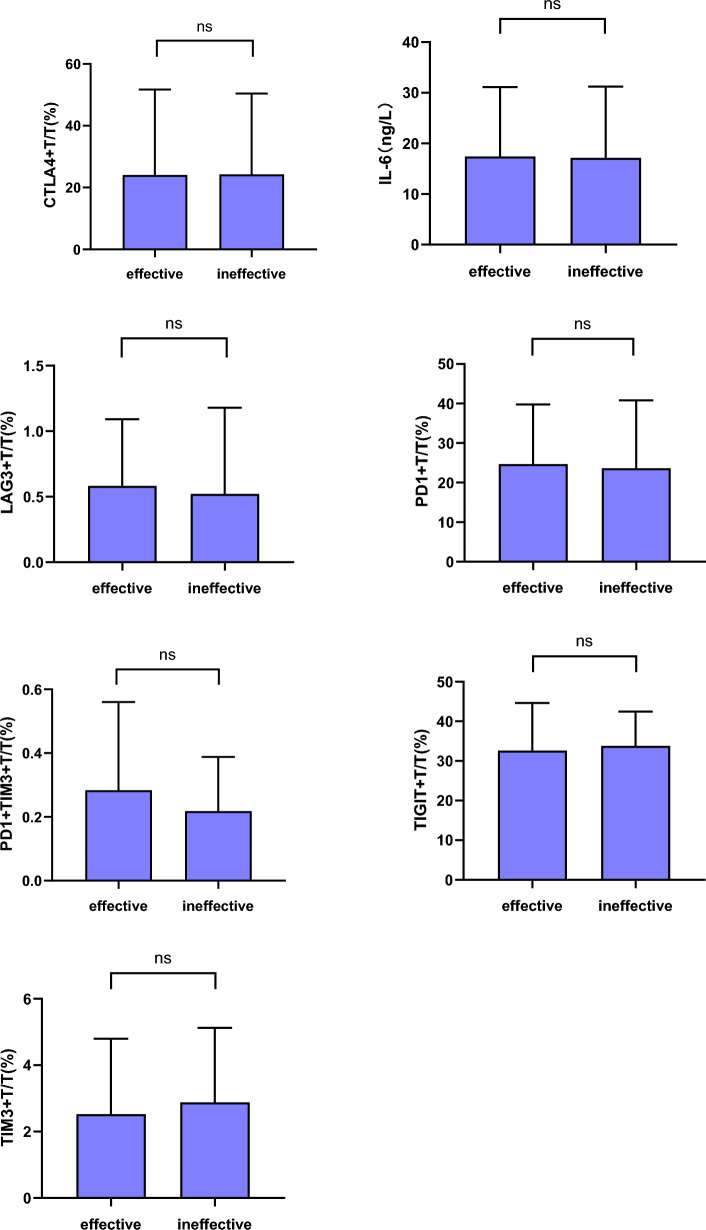
Comparison of lymphocyte subsets and IL-6 between the two groups of patients before immunotherapy (ns: *P* > 0.05).

Prior to immunotherapy, baseline levels of PD1 + TIM3+/T, LAG3 + T/T, IL-6, and PD1 + T/T in the effective group were higher compared to the ineffective group. Conversely, the TIM3 + T/T, CTLA4 + T/T, and TIGIT + T/T levels in the effective group were lower relative to the ineffective group. However, these baseline differences between the two groups were not statistically significant (*P* > 0.05), as shown in Fig. 1.

### Comparison of peripheral blood lymphocyte subsets and IL-6 after immunotherapy

**Figure 2:**
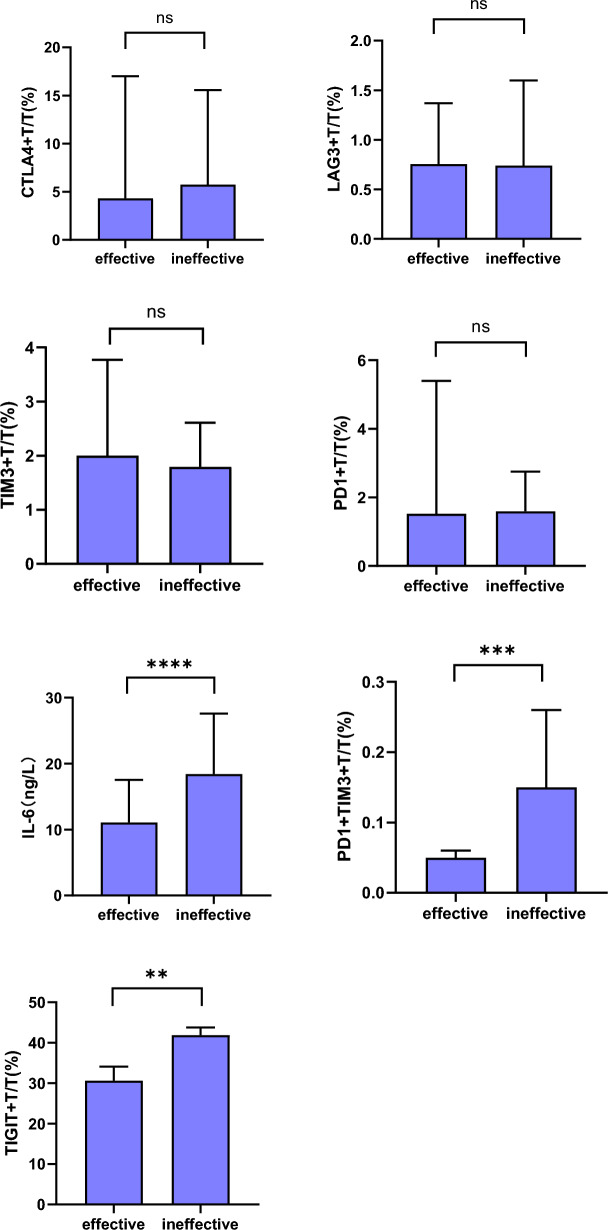
Comparison of peripheral blood lymphocyte subsets and IL-6 after immunotherapy. ns, *P* > 0.05; **, *P* < 0.01; ***, *P* < 0.001; and ****, *P* < 0.0001.

After immunotherapy, patients in the effective group showed lower levels of PD1 + TIM3+/T, TIGIT + T/T, and IL-6 than those in the ineffective group, with these differences being statistically significant (*P* < 0.05). For other lymphocyte subpopulations, no statistically significant difference were observed between the two groups (*P* > 0.05) (Fig. 2).

### Predictive ability analysis

**Figure 3:**
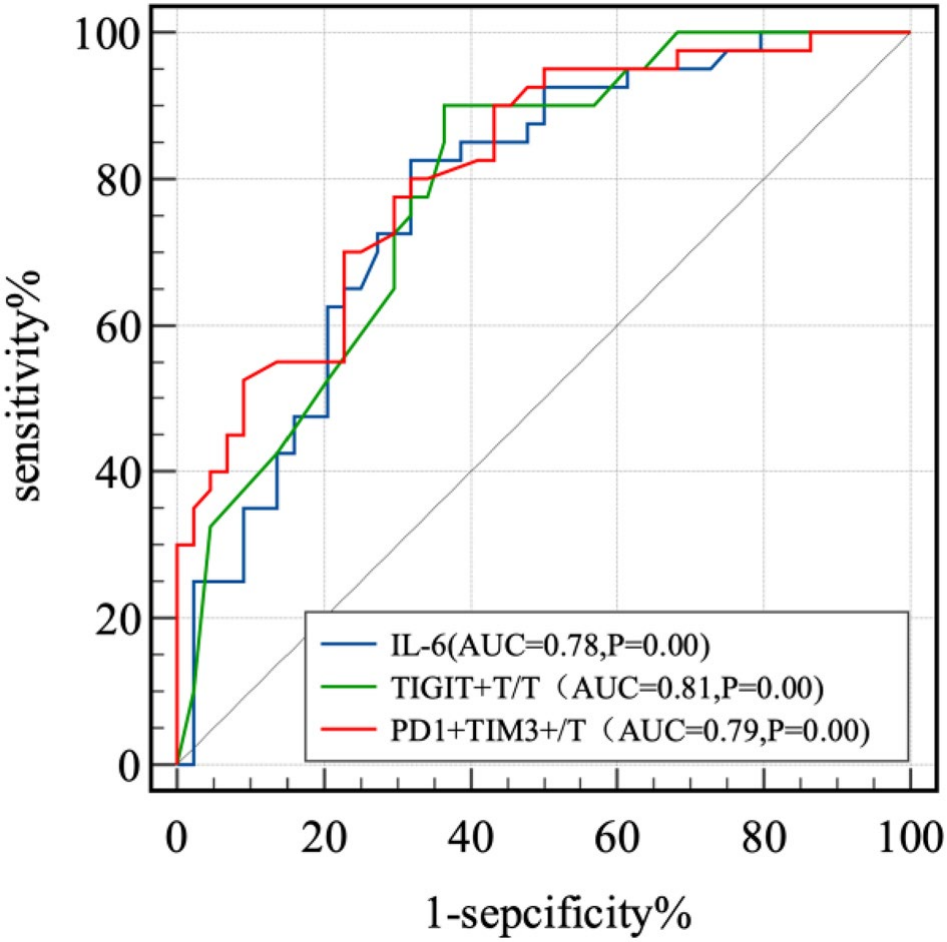
PD1 + TIM3 + T/T, TIGIT + T/T, IL-6 ROC curve.

From ROC analysis for PD1 + TIM3 + T/T, TIGIT + T/T, and IL-6, the optimal cutoff value was determined by the maximum Youden’s index (Fig. 3). Patients were grouped based on the cutoff value, and those above the cutoff value were divided into the high-value group, while those below the cutoff value were divided into the low-value group. The DCR was compared between the two groups.

**Figure 4:**
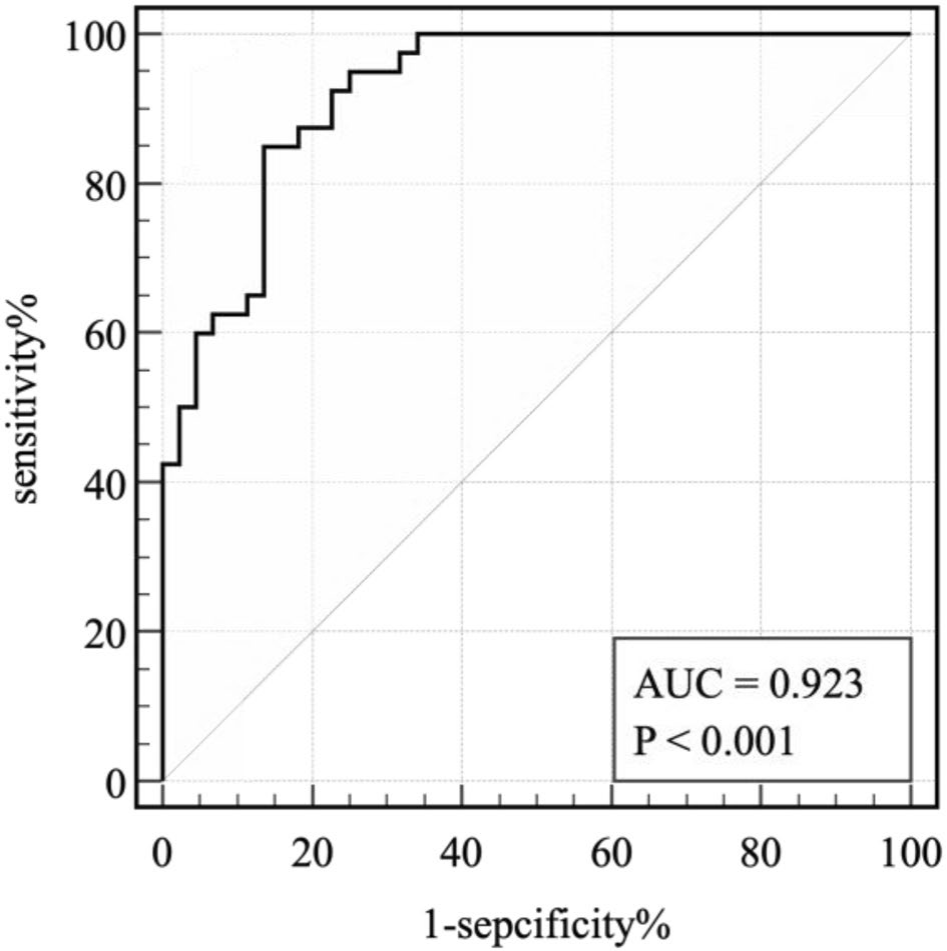
combined PD1^+^TIM3^+^T/T, TIGIT^+^T/T, IL-6 ROC curve.

The AUC for PD1 + TIM3 + T/T was 0.79 (95% CI 0.69–0.88, *P* < 0.0001), and the optimal cutoff value was 0.14% (sensitivity = 0.9, specificity = 0.64). For TIGIT + T/T was 0.81 (95% CI 0.73–0.90, *P* < 0.0001), and the optimal cutoff value was 35.14% (sensitivity = 0.82, specificity = 0.68), The AUC of IL-6 was 0.78 (95% CI 0.68–0.88 *P* < 0.0001), and the optimal cutoff value was 15.76 ng/L (sensitivity = 0.83, specificity = 0.68). Since PD1 + TIM3 + T/T/T, TIGIT + T/T, and IL-6 all had good predictive ability, we combined these three factors to derive a ROC curve, as shown in Fig. 4, with an area under the ROC curve (AUC) of 0.92 (sensitivity = 0.85, specificity = 0.86, *P* = 0.000).

These data indicated that after receiving immunotherapy, the levels of lymphoid subpopulations and IL-6 could effectively predict the treatment efficacy. Importantly, combining the three factors greatly improved the predictive value for efficacy. Furthermore, lower levels of PD1 + TIM3 + T/T/T, TIGIT + T/T, and IL-6 in the tumor microenvironment correlated with a potentially higher DCR, suggesting that patients have better clinical and survival benefits.

### DCR and PFS

After 2 cycles of immunotherapy, comparison of the DCR in the high-value and low-value groups of these three factors is shown in Table 3. Patients in the PD1 + TIM3 + T/T low-value group had a significantly higher DCR than those in the high-value group (72.5% vs. 9.1%, *P* = 0.000); patients in the TIGIT + T/T low-value group had a significantly higher DCR than those in the high-value group (65.2% vs. 26.3%, *P* = 0.000); and patients in the IL-6 low-value group had a significantly higher DCR than those in the high-value group. (53.8% vs. 32.4%, *P* = 0.04).

**Table 3:** Comparison of DCR between high and low value groups.

	PD1 + TIM3 + T/T				TIGIT + T/T				IL-6			
	High	Low	*X*^2^	*P*	High	Low	*X*^2^	*P*	High	Low	*X*^2^	*P*
CR	0	1			0	1			0	1		
PR	2	8			4	6			3	7		
SD	1	28			6	23			9	20		
PD	30	14			28	16			25	24		
DCR	9.1%	72.5%	32.3	0.00	26.3%	65.2%	12.6	0.00	32.4%	53.8%	12.04	0.04

**Figure 5:**
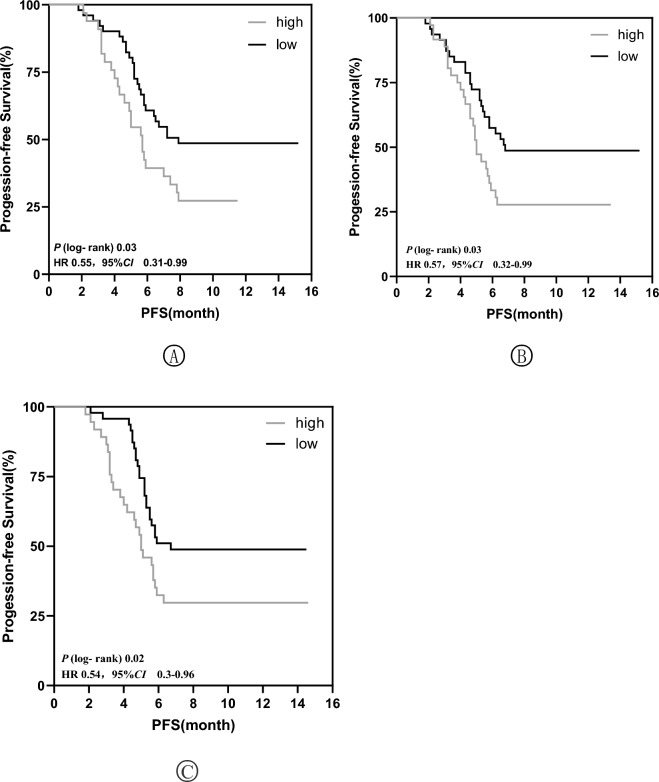
Progression-free survival curves stratified by PD1 + TIM3 + T/T (**A**), TIGIT + T/T (**B**), IL-6 (**C**) in HCC patients.

After 2 cycles of immunotherapy, comparison of the PFS in the high-value and low-value groups of these three factors is shown in Fig. 5. Patients in the PD1 + TIM3 + T/T low-value group had a significantly higher PFS than those in the high-value group (median, 8.9 vs. 5.70 months *P* < 0.03); stratified hazard ratio for progression, 0.55; 95% CI 0.31–0.99). patients in the TIGIT + T/T low-value group had a significantly higher PFS than those in the high-value group (median, 7.9 vs. 5.3 months *P* < 0.03); stratified hazard ratio for progression, 0.57; 95% CI 0.32–0.99); and patients in the IL-6 low-value group had a significantly higher PFS than those in the high-value group. (median, 7.8 vs. 5.2 months *P* < 0.02); stratified hazard ratio for progression, 0.54; 95% CI 0.3–0.96).

### Adverse events

No patients in this study experienced serious adverse reactions during the treatment period. When comparing the incidence of adverse events between the effective group and ineffective group, the effective group had a higher rate. However, this difference was not statistically significant (*X*^2^ = 3.086, *P* = 0.929) (Table 4).

**Table 4:** Treatment-related adverse events.

Adverse events	Effective (%)	Ineffective (%)
Hypertension	24 (26.7%)	25 (28.1%)
Proteinuria	20 (22.2%)	26 (29.2%)
Fatigue	4 (4.4%)	3 (3.4%)
Liver abnormalities	11 (12.2%)	4 (4.5%)
Rash	5 (5.6%)	3 (3.4%)
Thyroid dysfunction	8 (9.1%)	9 (10.1%)
Thrombocytopenia	2 (2.2%)	2 (2.2%)
Leukopenia	2 (2.2%)	1 (1.1%)
Bleeding	1 (1.1%)	2 (2.2%)
Diarrhea	2 (2.2%)	1 (1.1%)
Abdominal pain	2 (2.2%)	2 (2.2%)
Hand-foot syndrome	9 (10%)	11 (12.4%)

## Discussion

Although immunotherapy combined with tyrosine kinase inhibitors has shown significant promise in the treatment of advanced hepatocellular carcinoma, several clinical studies indicate that no more than 20% of patients benefit from PD-1 inhibitors alone^7,8^. Hence, there is an urgent clinical need to explore effective biomarkers that can identify patients likely to benefit from the treatment and guide personalized treatment strategies. Using genes to predict immunotherapy efficacy showed good accuracy in hepatocellular carcinoma patients reported at ASCO 2023^9^. For instance, a high TMB and low PLA2G44A expression level can predict a more favourable prognosis for patients receiving atelizumab plus bevacizumab. Similarly, low TP53 mutations and high CTNNB mutations can predict a better prognosis for patients receiving navulizumab. However, fewer studies investigated the role of immune status in predicting the efficacy of immunotherapy. In 2022, Chinese scholar Song Erwei^10^ first proposed the tumor ecology theory. Central to this theory is the classification of tumors into “cold” and “hot” tumors based on the degree of immune cell infiltration within the tumor. EICD (Effector immune cell deployment) is an important indicator for accurately defining cold and hot tumors^11^. Hence, by dynamically monitoring the immune status of the patients, we aim to more accurately predict the efficacy of immunotherapy.

T cells play a pivotal role in the anti-tumor immune response and can be divided into several distinct subpopulations and states. In chronic infectious or tumorigenic environments, T cells become exhausted due to persistent antigenic or T cell receptor (TCR) stimulation^12^. Further studies have shown that depleted T cells display the following shared features: impaired (but not absent) cytokine secretion, insufficient cytotoxicity, persistent high expression levels of multiple inhibitory receptors such as PD1, TIM3, LAG3, CTLA4, and TIGIT, decreased proliferative capacity, an altered transcriptional activities of the transcription factor TOX, and a unique epigenetic landscape. This depleted state often indicates a decreased tumor-killing capacity, which might significantly impact the efficacy of immunotheray^13–15^. In the past, PD1 expression served as a biomarker for predicting immunotherapy efficacy in various tumor types. However, recent findings suggest that PD1 may not adequately represent cellular dysfunction and exhaustion, underscoring the existence of more refined phenotypes and other surface receptors^16^. Thus, other lymphocyte subsets expressing immunosuppressive receptors could offer new avenues for investigation. Havel et al.^17,18^ found in a hepatocellular carcinoma mouse model that increased TIGIT expression represented T cell exhaustion and that TIGIT expression more reliably recognized exhausted T cells at different differentiation stages compared to PD-1. Moreover, TIGIT blockers synergistically inhibited carcinoma growth when combined with PD-1 inhibitors, suggesting that elevated expression of TIGIT may promote the progression of hepatocellular carcinoma. in patients with colorectal, gastric, renal, and leukemia^5,6,19,20^, PD-1 + Tim-3+ T cells produce fewer cytokines than other T cells types, resulting in diminished cytotoxicity and reduced proliferative capacity. It has also been found that increased IL-6 level is negatively correlated with the overall survival and progression-free survival of hepatocellular carcinoma patients^21–25^. However, the correlation between immune status and efficacy in hepatocellular carcinoma patients needs to be clarified.

Thus, in this study, various lymphocyte subpopulations expressing immunosuppressive receptors and IL-6 were selected as predictors of efficacy in hepatocellular carcinoma patients receiving immunotherapy. Our results showed that baseline lymphocyte subsets (PD1 + TIM3 + T/T, TIGIT + T/T, TIM3 + T/T, CTLA4 + T/T, LAG3 + T/T, PD1 + T/T) and IL-6 levels before immunotherapy did not affect treatment efficacy; whereas, after receiving 2 and more cycles of treatment, patients in the effective group showed significantly lower peripheral blood PD1 + TIM3+/T, TIGIT + T/T, and IL-6 than those in the ineffective group. This suggests that lymphocyte subsets and IL-6 after immunotherapy may serve as important valuable indicators for predicting efficacy. ROC analysis revealed a high predictive value for each of the three indicator. When combining the three indicators, the predicitive value for efficacy achieved a robust 0.93. Further, when patients were grouped based on the optimal cutoff value of PD1 + TIM3+/T, TIGIT + T/T, and IL-6, the DCR was higher in the low-value group compared to the high-value group.

Studies have demonstrated that PD-1 can bind to galectin-9 (Gal-9), a ligand for TIM-3. This binding impairs the Gal-9/TIM-3-induced PD-1 + TIM-3+ T cell death and persist depleted T cells, contributing to the development of hepatocellular carcinoma^26^. This suggests that PD-1 + TIM-3+ T cells depletion positively correlates with worse prognosis. It has also been found^27^ that in a mouse model of melanoma, PD-1 + Tim-3 + CD8+ T acquires its myeloid markers from the surface of antigen-presenting cells by trogocytosis, and is subsequently recognized and killed by T cells, suggesting a higher presence of cells with this phenotype can augment tumor burden and shorten survival time. On the other hand, TIGIT can compete with CD226 for binding to CD155, which results in increased IL-10 secretion and decreased IL-12 secretion due to TIGIT’s higher affinity of CD15. Consequently, dendritic cell-mediated immune response was attenuate, which further suppressed T-cell activity. TIGIT also directly inhibits downstream signaling pathways, such as PI3K and MAPK, through its intracellular ITIM structural domain, and transmits inhibitory signals internally to T cells and NK cells. In TIGIT+ Treg cells, the expression level of many immunosuppressing marker genes, including Foxp3, Helios, neuropilin-1, CTLA-4, PD-1 and LAG-3, was upregulated, which inhibited the tumor killing function of T cells^28–31^. Such findings suggest that high TIGIT expression is associated with a suppressed state immune microenvironment, potentially diminishing the efficacy of immunotherapy. In addition, IL-6 binding with its receptor forms a complexity. This complex then binds to the membrane protein gp130, assembling into a hexamer that triggers downstream signaling and gene expression. The downregulation of IL-6 expression can inhibit tumorigenesis and progression via the JAK-STAT3, RAS-RAF, and PI3K/Akt pathways^32–34^.

In conclusion, we demonstrated that PD1 + TIM3+/T, TIGIT + T/T, and IL-6 levels after immunotherapy can serve as predictors of efficacy in patients with hepatocellular carcinoma. T-cell depletion is influenced a combination of several suppressive factors and a higher proportion of suppressive lymphocyte subset correlates with reduced immunotherapy.

This study has several limitations. Firstly, the sample size was small and the follow-up duration was short. Future studies with the sample size should be enlarged and longer follow-up time to are needed to further validate the difference between progression-free survival time and overall survival. In addition, the expression of inhibitory receptors by depleted T cells is a time-heterogeneous step-by-step expression process. The time interval for blood sampling will be optimized to further explore the related mechanisms in future studies. We hope that we can provide patients with precise and individualized treatment in the future.

## Data Availability

The datasets generated and/or analysed during the current study are not publicly available due to it is a prospective study and follow-up is ongoing but are available from the corresponding author on reasonable request.
